# Flywheel resistance training in football: a useful rehabilitation tool for practitioners

**DOI:** 10.3389/fspor.2024.1434995

**Published:** 2024-07-05

**Authors:** Paolo Perna, Kevin L. de Keijzer, Marco Beato

**Affiliations:** ^1^ School of Allied Health Sciences, University of Suffolk, Ipswich, United Kingdom; ^2^ Medical Department, Chelsea Football Club, London, United Kingdom

**Keywords:** soccer (football), eccentric, prevention, return to play, hamstrings

## Introduction

Injuries in football are a common occurrence, given the sport's physically demanding nature. Previous research has shown that a team of 25 players can suffer around 50 injuries during a season, with players averaging two injuries per year (1). The most common injuries in football occur in order of incidence to the thigh, knee, and ankle (2). These injuries can occur due to different mechanisms of injury with direct contact injuries being more frequent than overuse injuries (2). Injuries can sideline players for a prolonged period of time and can negatively impact team performance (3), as well as clubs' finances (4). It is therefore paramount that players return from injury in the most effective way. An appropriate rehabilitation process is crucial for returning to optimal performance and reducing the likelihood of re-injury in football.

The rehabilitation program should involve members of the interdisciplinary team (e.g., medical, performance, and technical staff) and may involve external stakeholders (e.g., surgeons, consultants) at different stages to optimize the return to play (RTP) process. Throughout rehabilitation, the physiotherapist's role is pivotal in managing pain, restoring function (mobility, strength), and establishing expectations (timeframes, targets). After the acute phase (e.g., managing pain, improving mobility), the gym-based RTP program aims to restore strength (alongside other key performance parameters) to pre-injury level (or at least reduce the existing gap). A key component of a successful RTP program is the appropriate and timely integration of strength training (5). Strength training aims to gradually rebuild musculotendinous strength to ensure the player can cope with the physical demands of on-pitch training and matches upon return while minimizing risk of re-injury.

Flywheel training is an innovative training method used to develop strength in injured and healthy populations alike. A key aspect of flywheel training is the potential to achieve maximal voluntary forces throughout each repetition. To initiate movement, the user must pull or push a cord/strap attached to a fixed shaft [on which the flywheel disc(s) are placed]. The force applied unwinds a strap connected to the shaft of the devices, which starts to rotate. After the entirety of the desired concentric phase is performed, the cord/strap rewinds, and the user must resist the pull of the rotating flywheel disc(s) by performing an eccentric muscle action. If performed with appropriate technique and equipment, a greater application of force during the eccentric action allows for a mechanical eccentric overload and, thereby, greater muscle activation and mechanical power production. The eccentric power outputs are related to the strength applied during the concentric phase. The pairing of maximal concentric and eccentric efforts has been shown to enhance hypertrophy and strength as well as other physical parameters (6).

Considering that the evidence around the use of flywheel resistance training in RTP programs in football is very limited; this commentary aims to provide a rationale for the use of flywheel training and propose exercises to practitioners that can be integrated into RTP programs.

## Physical demands of football and return to play

Football demands a high level of physical fitness and endurance. Players often cover 9–14 km during a 90-min match, with midfielders and fullbacks running the most (7). The game is characterized by intermittent bursts of high-intensity activity interspersed with periods of lower intensity (between 1,200–1,400 changes of action), requiring players to have a well-rounded athletic profile (7, 8). In addition to the cardiovascular capacity to maintain a high level of performance throughout the game, players must have adequate strength and power levels to perform sport-specific activities such as jumping, tackling, changing direction, and sprinting (9). The knowledge of the physical demands of the sport is not only needed for coaches and sports scientists to prepare the players for the demands of the game but also for the practitioners who manage the rehabilitation process. RTP programs follow a phased progression with graded exposure to pitch-based activities to ensure that the players can complete all the necessary football actions before returning to training, competition and performance (10). This systematic and data-driven approach can also help reduce the likelihood of re-injury (11).

## Strength training and injury risk

Injuries in professional football are a significant concern due to their frequency and impact on players' careers and teams' performances. Specifically, re-injuries during the RTP process can profoundly impact players physically and psychologically while disrupting tactics, reducing competitiveness, and result in financial losses for teams (4). In an attempt to reduce the likelihood of sustaining injuries as well as re-injuries during the RTP, performance and medical departments need to implement prevention strategies. For instance, neuromuscular exercises and warm-up protocols can reduce the likelihood of acute non-contact injuries (12). Obviously, to prevent is better than to cure, and this adage holds true in professional football and during the RTP process.

Previous evidence suggests that various forms of strength training and in particular eccentric training can effectively lower injury risk amongst football players (13). Multi-faceted training regimens that merge strength work with balance and plyometric exercises have been shown to decrease the incidence of non-contact injuries (14). However, eccentric training, distinguished by its unique physiological benefits over other types of resistance training, is gaining support within the sports research community. Eccentric strength training has the ability to create changes in the muscle architecture (fascicle lengthening, pennation angle and muscle thickness) and improve eccentric hamstring strength which positive affect the HSI modifiable risk factors (15). For example, the Nordic hamstring exercise (NHE), which is an exercise used for strengthening the hamstrings and reducing the likelihood of injury, is quite a cost-effective strategy. During a NHE the players starts in a kneeling position with their ankles secured. They then lower their upper body forward, maintaining a straight line from head to heels, using only their hamstrings to control the descent. However, NHE has some important limitations: first, managing intensity progression can be challenging, second, the knee angles (ranging from 90° to 60° knee flexion) used during the exercise are not specific to the angles commonly associated with hamstring injuries in football and other team sport (16). Recently, flywheel resistance training has emerged as another valid method to obtain sport specific improvements and morphological adaptations. Flywheel resistance training uniquely combines concentric and eccentric muscle contractions, also shows promise in injury reduction. A recent paper by Beato and Dello Iacono (17), suggested that strength and conditioning professionals are encouraged to incorporate these strength training practices and eccentric-based exercises such as flywheel training into their weekly routines to help reduce injury likelihood—these recommendations could be extended to the RTP process to decrease the likelihood of muscle injuries (17).

## Flywheel resistance training during the return to play program

Flywheel training is a valuable tool during the RTP phase because it allows for a great deal of versatility through the customization of exercise intensity, volume, and technique (18). Unlike traditional isotonic training, flywheel training provides variable resistance throughout the movement, providing challenges when the muscles are actively lengthening under tension. This unique resistance profile may lead to neuromuscular responses and morphological adaptations (19). Specifically, these include greater motor unit discharge rates and selective recruitment of higher-order motor units, leading to improved muscle synchronization and strength. Overall, evidence supports the use of flywheel training to enhance strength, power, hypertrophy, and tendon stiffness (20). These adaptations are crucial for football players as they can enhance performance in terms of speed, jump, change of direction, and resilience to injury (19, 20). The versatility of flywheel devices allows for a wide range of exercises targeting different muscle groups and movement patterns relevant to the sport. In this paper, we have focused our attention on flywheel resistance exercises that can be included in a gym-based RTP program.

### Training the change of direction movements

It is well known that football requires multidirectional actions such as change of direction (COD) movements (21). The ability to effectively change direction and quickly react are key performance indicators. In football, high-intensity accelerations and decelerations have a positive impact on teams' performance outcomes (22) and are performed in important actions of the game, often before a goal is scored (23). Both accelerations and decelerations expose players to high physiological and mechanical stress levels. These high-demand actions have shown a correlation with the development of post-match neuromuscular fatigue (24, 25). The sudden braking activity during deceleration is controlled by an eccentric muscle contraction, which can generate higher mechanical forces than concentric or isometric contractions. Typically, in football players must decelerate their body mass (eccentric phase), control the joint movements, and re-accelerate (concentric action).

**Figure 1 and 2:**
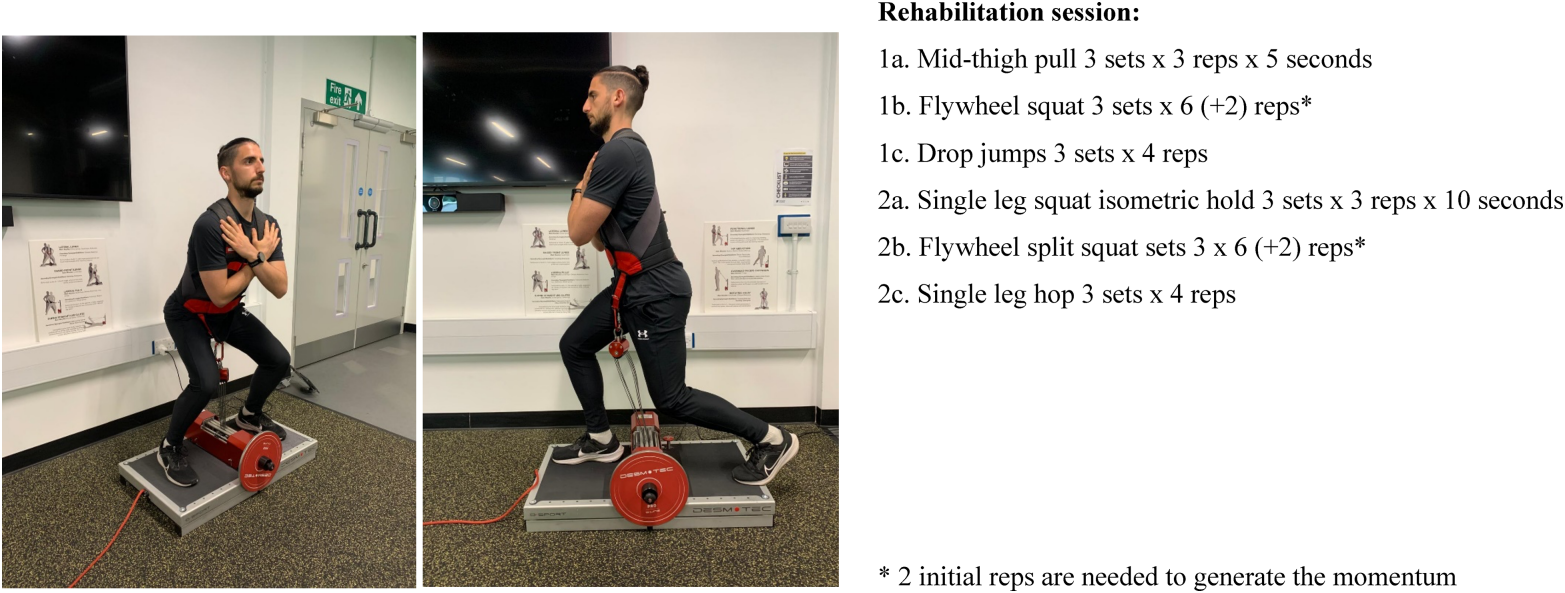
Flywheel squat and flywheel split squat..

The role of the quadriceps muscle to absorb forces and control the knee movements on different planes can also have a positive effect on injury prevention, in particular, controlling the rotation and valgus stress in the anterior cruciate ligament (ACL) injuries (26). Currently, there is no consensus on the best type of training to improve eccentric muscle capacity for deceleration actions, but flywheel resistance training has shown promising results (27). A systematic review highlighted the efficacy of flywheel resisted training in improving COD performance the similarities between COD tasks and flywheel training (6). Another study on under-19 footballers analyzed the effect of a 10-week flywheel training on the following kinetic parameters of COD: peak braking and relative propulsive force, contact time, time spent during braking and propulsive phase, relative total impulse and relative braking and propulsive impulse, and found substantial effects in several kinetic variables during COD tasks (28). Similarly, 11 weeks of strength training including flywheel exercises such as diagonal trunk rotations, backward lunges, unilateral hamstrings “kicks” and lateral squats improved COD ability measured with a V-cut test in young male football players (29). As reported above, the aim of the RTP is to prepare players to return to football at pre-injury level and with minimal risk of re-injury. When working with athletes who sustained an injury with a deceleration mechanism or are at risk of re-injury because of a braking mechanism, it is essential to expose and overload those movements before they return to football-specific training. Examples of exercises and prescriptions are in Figures 1, 2, videos in the Supplementary Material (Video S1, Flywheel squat and Video S2, Flywheel split squat).

### Hamstring injuries rehabilitation

Practitioners working in football can expect to face around eight hamstring muscle strain injuries (HSI) in their teams during a competitive season, and despite the effort and the research in injury prevention, the incidence of these injuries is increasing (30). Many studies have analyzed the most common mechanisms of injury and divided them into two main categories: sprinting and stretching, with the first one being more frequent (31). These injuries occur during sprinting-type (speed >25 km·h^−1^) (32) and closed-chain stretching-type movements. In both cases, the hamstring muscles are required to quickly contract eccentrically either during the late stance phase of the running gait or the braking movement to decelerate (16). Therefore, eccentric exercises are routinely used in rehabilitation to prepare the hamstring muscles to cope with the high force demands of sprinting. Practitioners could therefore use flywheel resistance training exercises since they showed promising results in developing hamstring strength, hypertrophy and creating fascicle length adaptations.

**Figure 3 and 4:**
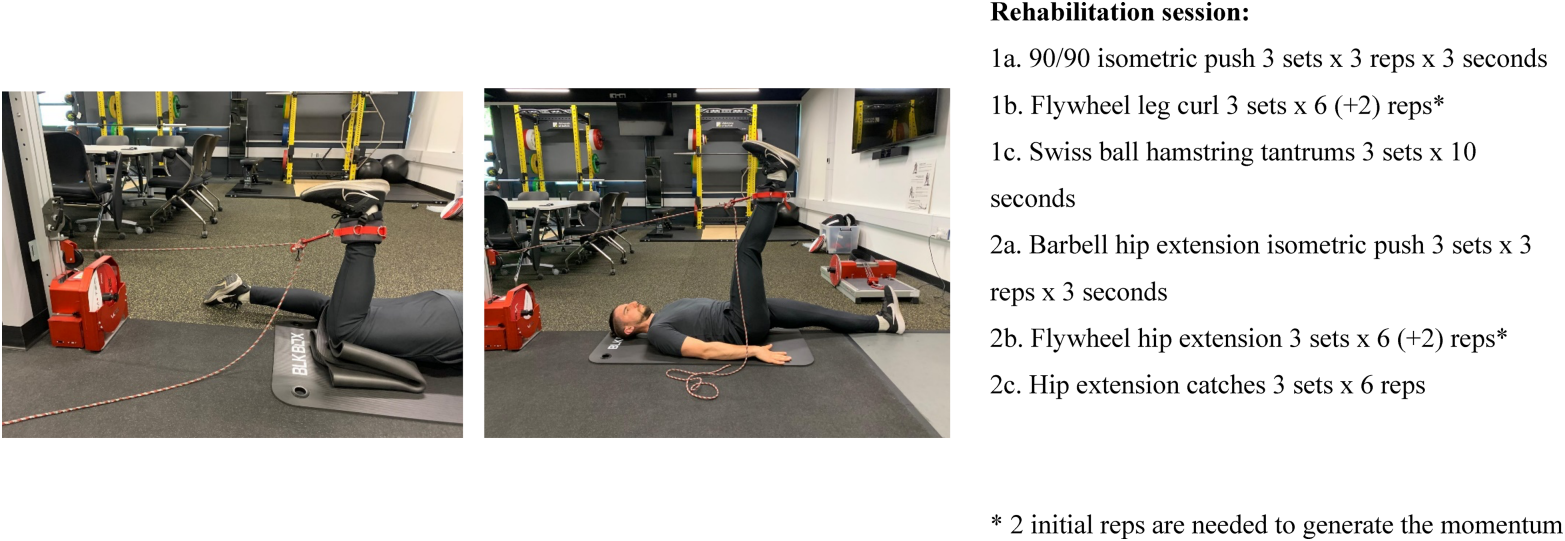
Flywheel Leg curl and flywheel Hip extension

Another factor to consider when prescribing hamstring strength exercises during the rehabilitation process is the specificity of the exercises based on muscle activation. In football, the majority of hamstring injuries are at the biceps femoris (84%), with the semitendinosus (11%) and the semimembranosus (5%) less frequently involved (33). The proximal area of the biceps femoris muscle is the most affected, and practitioners should be aware of the exercises that can target this area of the muscle and select them at appropriate times during rehabilitation. This is even more important considering that biceps femoris neuromuscular activation is often suppressed on the injured side compared to the uninjured side (34). Several studies have shown that exercises that involve the hip extension function of the hamstring muscles can activate more the proximal area of the biceps femoris: 45° hip-extension exercise, straight-knee bridge, unilateral and bilateral stiff-leg deadlift and hip hinge (35); hip extension conical pulley (36); straight-knee bridge, upright hip extension conic-pulley (37). Examples of exercises and prescriptions are in Figures 3, 4, videos in the Supplementary Material (Video S3, Flywheel leg curl and Video S4, Flywheel hip extension).

### Flywheel exercise prescription during rehabilitation

Due to the high energy demands and eccentric overload, flywheel resistance training is an exercise modality that is more prevalent in the late stages of gym-based rehabilitation. This idea was confirmed by a recent survey where the surveyed therapists preferred to use flywheel training in the late stages rather than the early stages of rehabilitation (38). This idea of progressively loading the injured structure using different exercise typologies aligns with the traditional rehabilitation model (39). In recent years, this traditional progression and delayed use of eccentric exercise has been reviewed. Some authors have proposed that an earlier introduction of eccentric training would not cause complications and could be performed safely (40, 41). The evidence is still lacking to support this theory fully, and the type of injury also plays a role in determining if an early eccentric load is appropriate. For example, hamstring intramuscular tendon injuries respond well to a rehabilitation program with delayed lengthening and eccentric activation (42, 43). Several studies have discussed the appropriate exercise prescription for flywheel training. Beato and Dello Iacono (17) reported as consistent in the literature, the use of multiple sets (from 3 to 6) and repetitions (from 6 to 8) (17), but there is also evidence that a higher number of sets (5) and repetitions (10) can produce earlier changes in muscles cross-sectional area when using flywheel-squat (44) in line with recent research on the correlation between training volume and hypertrophy (45). A similar number of sets and repetitions improved eccentric hamstring peak power using the flywheel leg-curl and flywheel hip-extension (46) and improved COD using the flywheel leg-curl and half squat (28).

When designing flywheel training sessions, it is important to consider the specific needs of the sport and the athlete. The most common errors in flywheel training include simply replacing traditional exercises with their flywheel counterparts or adding flywheel exercises without a clear purpose. Instead, practitioners should focus on integrating flywheel exercises that complement the athlete's training program and address their individual rehabilitation and performance goals. Although eccentric-based exercises can effectively activate the hamstring muscles, a recent study has highlighted that no exercise can reproduce a muscle activity close to sprinting, not even the NHE, the most widely utilized eccentric exercise (47). For such a reason, we recommend practitioners include sprint training not only for performance reasons but also to improve hamstring strength and prevent injuries during rehabilitation (48). Although several studies support the ability of flywheel training to create an eccentric overload (49), a systematic review concluded that eccentric overload is not always achieved when using flywheel devices but only when considering peak power and peak speed in the eccentric phase (50).

## Conclusions

Injuries in professional football are an unavoidable aspect of the sport, and a well-designed rehabilitation plan to bring players back to perform at the best of their abilities is paramount. Re-injuries are also frequently reported and one of the main goals during rehabilitation should be to reduce their incidence and severity. As research continues to evolve, it is hoped that the future of football will see fewer injuries and healthier, longer-lasting careers for its players. The collective efforts of players, coaches, medical professionals, researchers, and policymakers are essential in making the beautiful game safer for all involved. In conclusion, thanks to its ability to improve neuromuscular responses and morphological adaptations and create an eccentric overload, flywheel resistance training offers a unique and effective way to enhance athletic performance that can be utilized successfully during the rehabilitation process. Its science-based approach to resistance training can lead to significant improvements in strength, power, and overall athleticism.
